# Intraepidermal nerve fiber density as a predictor of cardiac events in Fabry disease

**DOI:** 10.1093/ehjopen/oeag042

**Published:** 2026-03-06

**Authors:** Kolja Lau, Dan Liu, Victoria Sokalski, Maximilian Gram, Kai Hu, Aljosha Lang, Christoph Wanner, Claudia Sommer, Nurcan Üçeyler, Peter Nordbeck

**Affiliations:** Department of Internal Medicine I, University Hospital Würzburg, Oberdürrbacher Str. 6, Würzburg 97080, Germany; Fabry Center for Interdisciplinary Therapy (FAZiT), University Hospital Würzburg, Oberdürrbacher Str. 6, Würzburg 97080, Germany; Department of Internal Medicine I, University Hospital Würzburg, Oberdürrbacher Str. 6, Würzburg 97080, Germany; Fabry Center for Interdisciplinary Therapy (FAZiT), University Hospital Würzburg, Oberdürrbacher Str. 6, Würzburg 97080, Germany; Department of Internal Medicine I, University Hospital Würzburg, Oberdürrbacher Str. 6, Würzburg 97080, Germany; Fabry Center for Interdisciplinary Therapy (FAZiT), University Hospital Würzburg, Oberdürrbacher Str. 6, Würzburg 97080, Germany; Department of Internal Medicine I, University Hospital Würzburg, Oberdürrbacher Str. 6, Würzburg 97080, Germany; Experimental Physics 5, University of Würzburg, Am Hubland, Würzburg 97074, Germany; Department of Internal Medicine I, University Hospital Würzburg, Oberdürrbacher Str. 6, Würzburg 97080, Germany; Fabry Center for Interdisciplinary Therapy (FAZiT), University Hospital Würzburg, Oberdürrbacher Str. 6, Würzburg 97080, Germany; Department of Neurology, University Hospital Würzburg, Josef-Schneider-Str. 11, Würzburg 97080, Germany; Fabry Center for Interdisciplinary Therapy (FAZiT), University Hospital Würzburg, Oberdürrbacher Str. 6, Würzburg 97080, Germany; Fabry Center for Interdisciplinary Therapy (FAZiT), University Hospital Würzburg, Oberdürrbacher Str. 6, Würzburg 97080, Germany; Department of Neurology, University Hospital Würzburg, Josef-Schneider-Str. 11, Würzburg 97080, Germany; Fabry Center for Interdisciplinary Therapy (FAZiT), University Hospital Würzburg, Oberdürrbacher Str. 6, Würzburg 97080, Germany; Department of Neurology, University Hospital Würzburg, Josef-Schneider-Str. 11, Würzburg 97080, Germany; Department of Internal Medicine I, University Hospital Würzburg, Oberdürrbacher Str. 6, Würzburg 97080, Germany; Fabry Center for Interdisciplinary Therapy (FAZiT), University Hospital Würzburg, Oberdürrbacher Str. 6, Würzburg 97080, Germany

**Keywords:** Fabry disease, Hypertrophic cardiomyopathy, Malignant arrhythmia, Device therapy, Intraepidermal nerve fiber density, Small fiber neuropathy

## Abstract

**Aims:**

Anderson-Fabry disease (FD) is a rare lysosomal storage disorder with multi-organ involvement. Cardiac manifestations are the major determinant of prognosis, yet reliable predictors of disease progression are lacking. Small fiber neuropathy can emerge in various aetiologies of hypertrophic cardiomyopathy, but its correlation remains unclear. Intraepidermal nerve fiber density (IENFD) from skin punch biopsy provides an established tool of measuring small fiber dysfunction. Clinically, we observed that FD patients with severe cardiac phenotypes often show markedly reduced IENFD without presentation of typical neurological symptoms matching small fiber neuropathy.

**Methods and results:**

FD patients with available skin biopsy samples evaluated between 2006 and 2022 were included. Patients were divided by median IENFD for clinical evaluation. Subsequent subgroup analysis for two predefined cardiac endpoints (development of late gadolinium enhancement (LGE) on cardiac MRI and cardiac device implantation) were done. Multivariable Cox regression analysis assessed the prognostic value of IENFD. A total of 170 patients (47% men) were analysed. Median IENFD was 3.8 fibers/mm. Patients with reduced IENFD showed more frequent cardiac (85% vs. 41%), renal (54% vs. 21%), and cerebrovascular (27% vs. 7%) involvement (all *P* < 0.001). FD cardiomyopathy was more advanced in those with low IENFD (IVSd: 12.5 ± 3.3 vs. 9.6 ± 2.3 mm, *P* < 0.001). In the conducted subgroup analysis IENFD < 4.2 fibers/mm independently predicted LGE (HR: 5.26, 95% CI: 1.53–18.06, *P* = 0.008, *n* = 55 patients included), while IENFD < 1 fiber/mm predicted device implantation (HR: 3.72, 95% CI: 1.00–13.78, *P* = 0.05, *n* = 124 patients included).

**Conclusion:**

IENFD was identified as independent risk factor for developing two major cardiac endpoints (LGE in MRI/device implantation) associated with poor cardiac outcome in FD.

**ClinicalTrials.gov Identifier:**

NCT03362164

## Graphical Abstract

**Graphical Abstract:**
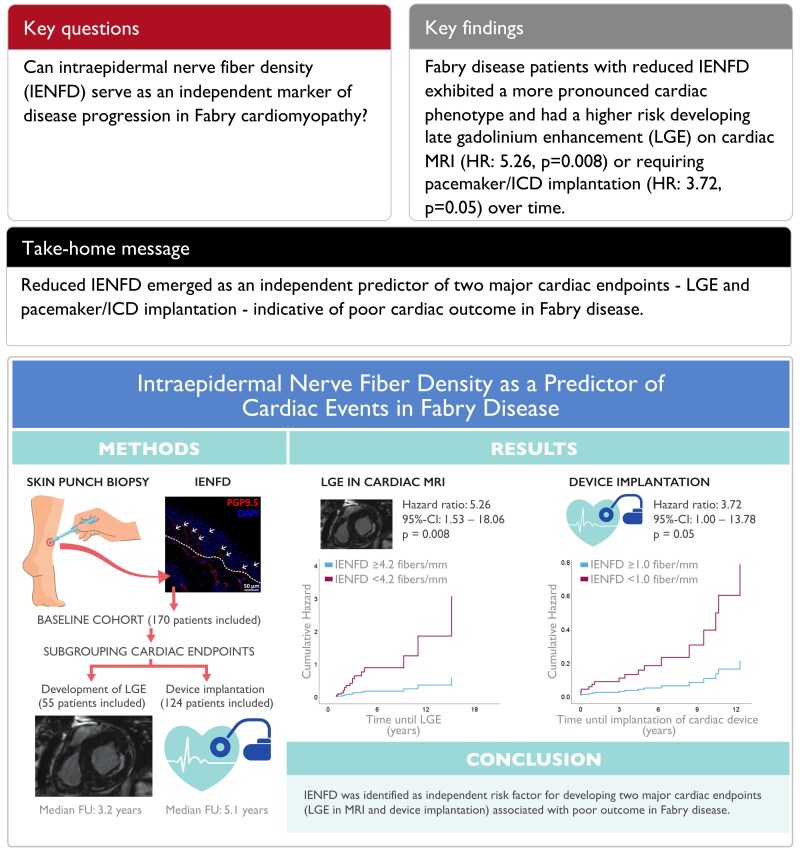
Intraepidermal nerve fiber density as a predictor of cardiac event in Fabry disease Long-term data reveal a novel association between small fiber neuropathy and cardiomyopathy in Fabry disease. Reduced IENFD independently predicts LGE on cardiac MRI and cardiac device implantation—both established markers of adverse outcome.

## Introduction

Anderson-Fabry disease (FD) is a rare lysosomal storage disorder with high symptom load in affected patients.^1,2^ X-linked genetic transmission lead to more severely affected male patients but several studies showed that females also develop FD-specific symptoms.^3,4^ Classic FD organ involvement that leads to a high disease burden, disability, and death includes kidney dysfunction, cerebrovascular events, and hypertrophic cardiomyopathy (HCM).^5^ Restrictions in everyday life also arise from neurological symptoms such as neuropathic pain. FD associated pain usually presents with a burning character and is mainly located in hands and feet, mostly starting in early childhood.^6–8^

Typical neurological symptoms, apart from acral burning pain, include thermal hypoesthesia or hypo- to anhidrosis.^9^ These symptoms are considered to represent dysfunction of small calibre A-delta and C nerve fibers, commonly referred to as small fiber neuropathy.^10^ In diagnostic assessments, quantitative sensory testing (QST) serves as a valuable tool for objectively evaluating symptoms.^11^ Histo-morphologically, FD patients regularly show a characteristic reduction of intraepidermal nerve fiber density (IENFD) in skin punch biopsies taken from distal and also proximal parts of the body.^12^ However, previous studies, trying to confirm FD diagnosis by assessing small nerve fibers in skin punch biopsies were unsuccessful.^13^ Diagnosing FD only by IENFD may ultimately not be possible since reduction of IENFD is not a disease-specific finding.^14^ Still, previous studies analysing clinical data of FD patients showed that results from skin punch biopsies correlate with disease severity in other affected organ systems.^9,12^

Cardiac involvement is the primary determinant of reduced life expectancy in FD patients.^15^ Cardiomyopathy is difficult to diagnose at early disease stages and usually progresses slowly at young age.^16^ The initial manifestations typically include non-specific electrocardiographic abnormalities, such as PR interval shortening or T-wave inversions, which are subsequently followed by progressive left ventricular hypertrophy (LVH).^17^ Hypertrophy is usually accompanied by myocardial fibrosis, measurable by late gadolinium enhancement (LGE) in cardiac MRI.^18^ End-stage FD cardiomyopathy results in heart failure with preserved or reduced ejection fraction due to extensive scarring of the myocardium. Besides these structural and functional changes, patients often suffer from malignant arrhythmias such as severe bradycardias and/or ventricular tachycardias (VTs), which can result in sudden cardiac death.^19^ Owing to its X-linked inheritance and the often incomplete manifestation of FD, particularly in women, early diagnosis can be challenging. This challenge is further compounded in late-onset variants, which may present with non-classical disease courses.^20^ Accurate predictions of whether a patient will benefit from specific medical or cardiac device therapy often remain an obstacle in clinical routine.^21^ Reliable markers to predict disease progression or adverse cardiac events are currently lacking.

In this work, we examined the association between IENFD and FD cardiomyopathy. Since FD pain manifests much earlier than cardiac or renal symptoms and is assumed to ground on small fiber neuropathy, this approach was considered particularly valuable for accurate stratification of prognosis and potentially also therapeutic guidance.

## Methods

Patients were included in a prospective, single-centre observational cohort study in Würzburg, Germany (clinicaltrials.gov Identifier: NCT03362164). Data were collected between 2006 and 2022. Inclusion criteria were positive genetic testing for FD, age ≥ 16 and available skin punch biopsy qualifying for histological analysis of IENFD. Subjects carrying benign/non-cardiac FD variants (*p.D313Y; p.A143T; p.S126G*) were excluded.^22–24^

**Figure 1:**
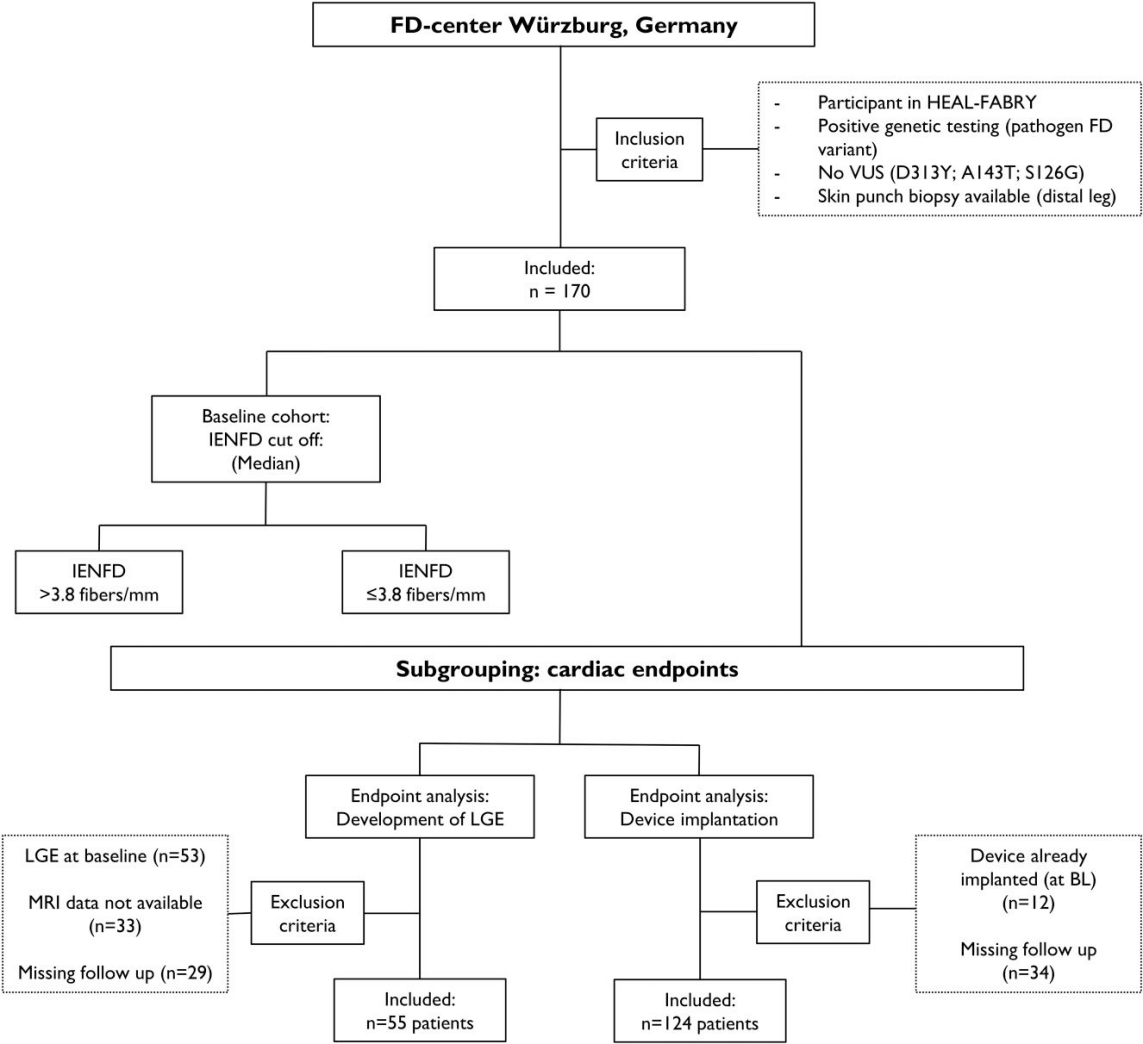
Flow chart illustrating patient’s distribution inclusion and exclusion criteria. A total of 170 patients were included in the baseline analysis. Subsequent subgroup analyses were conducted for two predefined cardiac endpoints. For the evaluation of LGE development, 55 patients were included, while 53 patients were excluded due to pre-existing LGE at baseline, 33 due to missing MRI data, and 29 due to lack of follow-up. Regarding the endpoint of device implantation, 124 patients were included; 12 patients were excluded because a device had already been implanted, and 34 patients were excluded due to missing follow-up data. Abbreviations: **BL** = baseline; **FD** = Fabry disease; **IENFD** = intraepidermal nerve fiber density; **LGE** = late gadolinium enhancement; **MRI** = magnetic resonance imaging **VUS** = variant of unknown significance.

This study analysed longitudinal data using two predefined major cardiac endpoints, which were assessed for their prognostic relevance. Baseline (BL) was defined as first visit when skin punch biopsy was performed. Patients were divided, into two groups based on sex and individual IENFD. Further explanations about defined endpoints and subgrouping are shown in *Figure 1* and below.

All patients received a comprehensive clinical evaluation, including a cardiac, nephrological, and neurological examination. Cardiac assessment included resting, exercise and Holter-ECG, echocardiography, and cardiac MRI, if possible and tolerated by the patient. Cardiac MRI protocol included cine imaging and LGE imaging after application of a contrast agent.

Laboratory parameters included NTproBNP and highly sensitive cardiac Troponin T (hs-cTnT).^25,26^ GFR (MDRD formula) and proteinuria (urine-albumin-creatinine-ratio) were measured for assessing kidney function.^27,28^ If clinically indicated, additional kidney biopsy was performed. Neurological examination included nerve conduction studies, QST, cerebral and spine MRI imaging, and skin punch biopsy at the lower leg and back to determine IENFD.

FD-specific biomarkers were measured at each visit. Alpha-galactosidase-A-enzyme activity was measured in leucocytes (normal range: 0.4–1.0 nmol/min/mg protein) and FD-specific biomarker Lyso-GB3 was measured in blood plasma (normal range: ≤ 0.9 ng/mL).^29–31^

### Skin punch biopsy and IENFD determination

**Figure 2:**
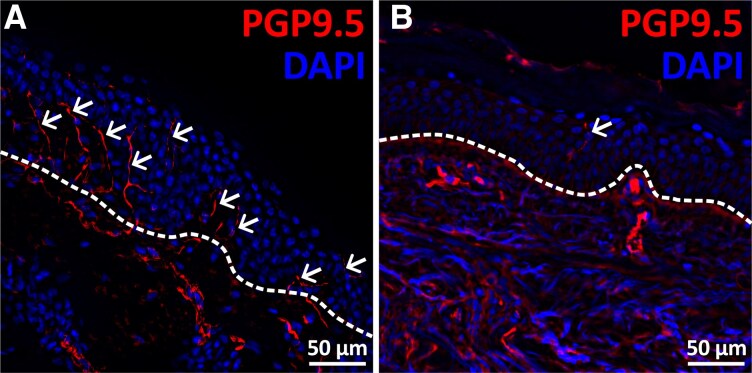
Immunohistochemical visualization of intraepidermal nerve fibers in human skin. Representative photomicrographs of skin sections exhibiting normal (*A*) and reduced (*B*) IENFD labelled with antibodies against PGP9.5 (red). Samples were counterstained with DAPI (blue) to visualize cell nuclei. The dashed white line indicates the epidermis-dermis border. White arrows mark individual intraepidermal nerve fibers. Scale bars: 50 µm. Abbreviations: **DAPI** = 4’,6-diamidino-2-phenylindole; **IENFD** = intraepidermal nerve fiber density; **PGP9.5** = protein gene product 9.5.

Two skin punch biopsies were taken from each patient under local anaesthesia (Stiefel GmbH, Offenbach, Germany). One proximal biopsy was taken from the back at level of thoracic vertebra 10 and the second one from the lateral distal leg. The processing of the biopsy specimen was performed according to previously established protocols.^20,22,32^ IENFD was determined by experienced investigators. Whether IENFD was reduced or in the normal range was assessed according to published rules.^33,34^  *Figure 2* shows a healthy control (A) and a highly pathological (B) result in comparison.

### Subgrouping and adverse cardiac events

Two cardiac endpoints were defined for analysing IENFD and their potential predictive value for the development of cardiac FD manifestations. The first endpoint was LGE in cardiac MRI. LGE was suggested to be a surrogate parameter for myocardial fibrosis in FD, which is linked with poor outcome.^35,36^ Previous studies demonstrated that FD patients might acquire myocardial LGE not only as a result but sometimes also prior to LVH.^37^ The second endpoint was the implantation of implantable cardioverter defibrillator (ICD) or pacemaker in FD patients due to malignant ventricular arrhythmias or severe bradycardia. These severe arrhythmias are one of the main reasons for sudden death in FD.^17^

For endpoint analysis, patients were stratified as outlined in *Figure 1*. The main reason for exclusion (endpoint: development of LGE) was LGE already described at baseline, missing MRI or follow-up data. In total, 55 patients were included for further analyses.

Reasons for exclusion for the second endpoint (cardiac device implantation) were already implanted cardiac devices (*n* = 12) or missing follow-up (*n* = 34). A total of 124 patients met the inclusion criteria for further analysis.

By not knowing optimal cut-off-values for IENFD in both endpoints, we determined the best thresholds for outcome prediction by utilizing the *Youden*-Index derived from ROC curves in both subgroups.

### Statistical analysis

Baseline characteristics were summarized after splitting the cohort by the median IENFD. Continuous variables are presented as mean ± standard deviation (SD) or median [interquartile range], and categorical variables as counts (%). Between-group differences were tested with the χ^2^ test or Fisher’s exact test for categorical data and the *t*-test or Mann–Whitney *U* test for continuous data, as appropriate. Normality was assessed using the Shapiro–Wilk test and visual inspection of Q–Q plots. Effect sizes for continuous comparisons were expressed as Cohen’s *d*.^38,39^

Time-to-event analyses were performed within the same parent cohort using endpoint-specific complete-case sets: (i) incident LGE in the MRI-available subset and (ii) cardiac device therapy (ICD or pacemaker implantation) in the full cohort with complete covariates. Survival functions were displayed with Kaplan–Meier curves and compared using the log-rank (Mantel–Cox) test. Univariable Cox proportional hazards models estimated hazard ratios (HRs) with 95% confidence intervals (CIs) for candidate predictors.

Multivariable analyses used forced-entry (enter) Cox models prespecified to adjust for age and sex, jointly evaluating IENFD and QST cold detection threshold (CDT). Endpoint-specific IENFD cut-offs were defined *a priori* from ROC/Youden analyses (LGE: < 4.2 vs. ≥ 4.2 fibers/mm; device therapy: < 1.0 vs. ≥ 1.0 fibers/mm).^40,41^ The proportional hazards assumption was assessed for the primary Cox regression models using time-dependent covariate interactions [covariate × log(time)].

Additional analysis for evaluating QST data (CDT) were shown in the supplementary appendix. To assess the incremental prognostic value of IENFD vs. QST CDT, we fitted nested block-entry Cox models in both directions: (Base A) age + sex + QST, then adding IENFD; and (Base B) age + sex + IENFD, then adding QST. Improvement in fit was quantified by the change in −2 log-likelihood (Δ−2LL) with 1 degree of freedom, and model parsimony was evaluated using AICc (small-sample corrected Akaike information criterion) and Bayesian information criterion derived from the model −2LL and the number of parameters. Collinearity between IENFD and QST was assessed using Spearman’s rank correlation and variance inflation factors (VIFs) from the multivariable Cox models; predefined thresholds (e.g. VIF < 2) were used to indicate negligible collinearity.^42^ Both markers were retained jointly when collinearity was not of concern.

For all statistical analysis, a *P*-value *≤*0.05 was assumed significant. All statistical analysis were performed by IBM SPSS version 29 for Windows (IBM, Armonk, New York, USA).

### Ethical aspects of this work

All patients were included in the HEAL-FABRY observational study (ClinicalTrials.gov Identifier: NCT03362164). Written informed consent was obtained from all participants after detailed explanation of the study’s objectives. The study adheres to the principles of the Declaration of Helsinki. Approval was obtained from the local ethics committee at University of Würzburg.

## Results

**Figure 3:**
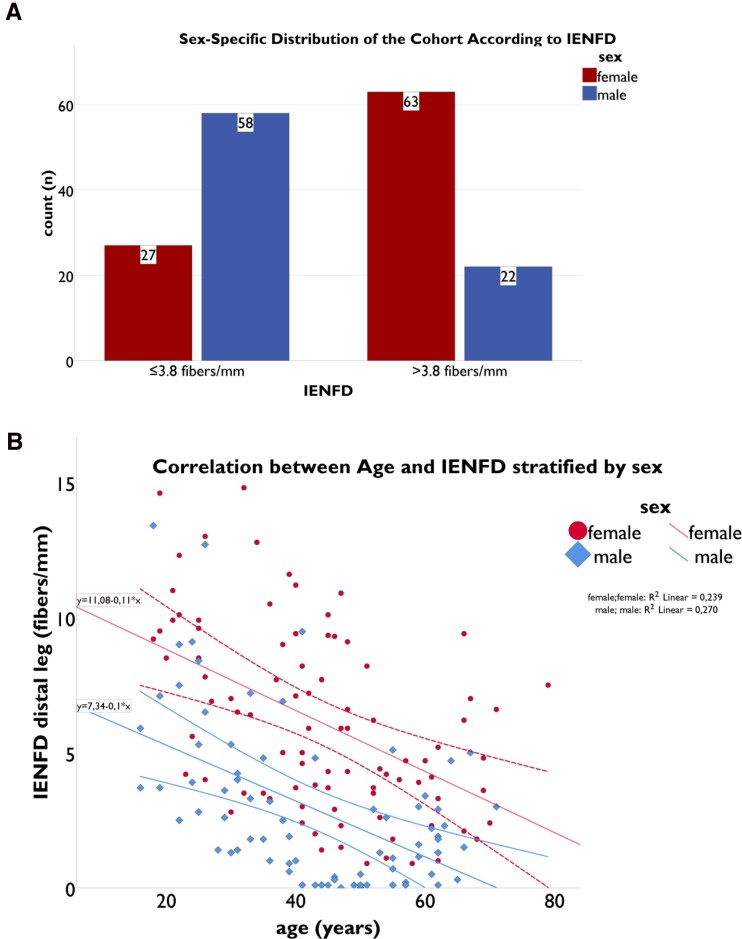
Distribution of patients in the baseline cohort. The upper panel (*A*) illustrates the distribution of patients within the cohort stratified by the median IENFD cutoff (> 3.8 fibers/mm). Red bars represent female patients, while blue bars represent male patients. The lower panel (*B*) depicts the correlation between IENFD and age for each individual patient. Male patients are shown in blue, and female patients in red. The lines represent linear regression models with 95% CI, demonstrating the relationship between IENFD and age stratified by sex. Abbreviations: **CI** = confidence interval; **IENFD** = intraepidermal nerve fiber density.

We included 170 patients in our analysis (80 men, 47%). In 119 patients (70%), a reduced IENFD in skin punch biopsy was reported. The identified genetic variants and their distribution among patients are presented in the supplementary appendix (Supplementary material online, *Table S4S*, Supplementary material online, *Figure S1S*). For baseline characteristics, the median was used to split the cohort into two groups defined by IENFD in skin punch biopsy at distal leg. The calculated median was 3.8 fibers/mm. *Figure 3A* shows the distribution of patients divided by sex and IENFD.

### Baseline characteristics

*Table 1* shows baseline characteristics of the investigated cohort, stratified for IENFD. Patients with reduced IENFD were more often male (68% vs. 26%, *P* < 0.001) and older (48 ± 13 vs. 39 ± 15, *P* < 0.001). Genetic variants were distributed equally. Manifestations of the heart, kidney and cerebrovascular events were more often seen in patients with reduced IENFD (heart: 85% vs. 41%; kidney: 54% vs. 21%; cerebrovascular events: 27% vs. 7%, for all *P* < 0.001). However, symptoms of the peripheral nervous system, described by the patients, showed no differences (any manifestation: 59% vs. 65%, *P* = 0.528; hyperhidrosis: 14% vs. 9% or hypo-/anhidrosis: 40% vs. 31%, *P* = 0.205, FD associated pain: 55% vs. 52%, *P* = 0.759). CDT assessed by QST showed significant differences with CDT being worse in patients with reduced IENFD (CDT: −10.09 vs. −5.17, *P* < 0.001). LVH and mass were higher in patients with reduced IENFD (IVSd: 12.5 ± 3.3 mm vs. 9.6 ± 2.3 mm, *P* < 0.001; Diastolic mass (BSA) in MRI: 95.2 ± 36.3 g/m^2^ vs. 71.9 ± 20.7 g/m^2^, *P* < 0.001). Additionally, LGE was observed with greater frequency (37% vs. 26% *P* = 0.013) and an already implanted cardiac device occurred more commonly in patients with reduced IENFD (12% vs. 2%, *P* = 0.032).

**Table 1:** Baseline characteristics

Parameter		All patients (*n* = 170)	IENFD ≤ 3.8 (*n* = 85)	IENFD > 3.8 (*n* = 85)	*P*
**Male sex [*n* (%)]**		80 (47%)	58 (68%)	22 (26%)	**<0.001**
**Age [years]**		44 ± 15	48 ± 13	39 ± 15	**<0.001**
**(Classic) missense variants [*n* (%)]**		71 (42%)	34 (40%)	37 (44%)	0.853
**(Classic) nonsense variants [*n* (%)]**		58 (34%)	29 (34%)	29 (34%)	
**Late onset variants (p.N215S) [*n* (%)]**		41 (24%)	22 (26%)	19 (22%)	
**Organ involvement**					
**Heart [*n* (%)] (any signs of disease)**		107 (63%)	72 (85%)	35 (41%)	**<0.001**
**Kidney [*n* (%)] (KDIGO stage G1A2 and lower)**		64 (38%)	46 (54%)	18 (21%)	**<0.001**
**Cerebrovascular [*n* (%)] (TIA or stroke)**		29 (17%)	23 (27%)	6 (7%)	**<0.001**
**Any symptoms of peripheral nervous system [*n* (%)]**		105 (62%)	50 (59%)	55 (65%)	0.528
	*Hyperhidrosis* [*n* (%)]	20 (12%)	12 (14%)	8 (9%)	0.205
	*Hypo−/anhidrosis* [*n* (%)]	60 (35%)	34 (40%)	26 (31%)	
	*FD pain* [*n* (%)]	91 (54%)	47 (55%)	44 (52%)	0.759
**QST cold detection threshold (value)**		−7.64 (*n* = 169)	−10.09	−5.17 (*n* = 84)	**<0.001**
**QST cold detection threshold (z-score)**		−0.65 (*n* = 169)	−1.08	−0.22 (*n* = 84)	**<0.001**
**GI- symptoms [*n* (%)] (any reported symptoms)**		59 (35%)	29 (34%)	30 (35%)	1
**Cornea verticillata [*n* (%)] (ophthalmologic diagnosed)**		54 (32%) (*n* = 143)	25 (29%) (*n* = 69)	29 (34%) (*n* = 74)	0.733
**ENT [*n* (%)] (any reported symptoms)**		92 (54%)	53 (62%)	39 (46%)	**0**.**045**
**Angiokeratoma [*n* (%)]**		49 (29%)	37 (44%)	12 (14%)	**<0.001**
**Ongoing FD-specific therapy or indication seen at BL [*n* (%)]**		114 (67%)	70 (82%)	44 (52%)	**<0.001**
***FD therapy at baseline* [*n* (%)]**		40 (24%)	30 (35%)	10 (12%)	**<0.001**
***FD therapy indication seen at baseline* [*n* (%)]**		74 (44%)	40 (47%)	34 (40%)	0.439
**Skin biopsy**					
**Reduced IENFD reported [*n* (%)]**		119 (70%)	85 (100%)	34 (40%)	**<0.001**
**IENFD distal leg [fibers/mm]**		4.6 ± 3.6	1.7 ± 1.2	7.5 ± 2.8	**<0.001**
**IENFD back [fibers/mm]**		20.6 ± 9.2 (*n* = 167)	16.33 ± 8.4 (*n* = 82)	24.8 ± 8	**<0.001**
**Cardiac/Kidney/FD parameters**					
**IVSd—echocardiography [mm]**		11.1 ± 3.2	12.5 ± 3.3	9.6 ± 2.3	**<0.001**
**LVPWd—echocardiography [mm]**		10.5 ± 2.7	11.4 ± 2.8	9.5 ± 2.3	**<0.001**
**A-Gal enzyme activity in leucocytes [nmol/min/mg protein]**		0.17 ± 0.15 (*n* = 160)	0.11 ± 0.13 (*n* = 80)	0.23 ± 0.15 (*n* = 80)	**<0.001**
**Lyso-Gb3 [ng/mL]**		32.6 ± 49.2 (*n* = 157)	46.8 ± 57.3 (*n* = 80)	17.9 ± 33.7 (*n* = 77)	**<0.001**
**GFR (MDRD) [mL/min/1.73 qm]**		87.1 ± 29.9 (*n* = 168)	73.6 ± 28.3 (*n* = 83)	100.3 ± 25.4	**<0.001**
**Cystatin C [mg/L]**		1.05 ± 0.81 (*n* = 163)	1.3 ± 1.07 (*n* = 80)	0.81 ± 0.26 (*n* = 83)	**<0.001**
**UACR [mg/g creatinine]**		229.3 ± 591.1 (*n* = 162)	404.2 ± 791.3 (*n* = 77)	70.9 ± 222.8	**<0.001**
**Troponin (hs) [pg/mL]**		31.9 ± 70.4 (*n* = 89)	49.2 ± 91 (*n* = 49)	10.6 ± 12.4 (*n* = 40)	**<0.001**
**NT-proBNP [pg/mL]**		533.5 ± 974.1 (*n* = 163)	822.9 ± 1207.6 (*n* = 78)	267.9 ± 586.6	**<0.001**
**Ongoing dialysis [*n* (%)]**		5 (3%)	5 (6%)	0 (0%)	0.059
**Kidney transplantation [*n* (%)]**		1 (1%)	1 (1%)	0 (0%)	1
**MRI parameters**					
**Data available [*n* (%)]**		137 (81%)	61 (72%)	76 (89%)	**0**.**006**
**Diastolic mass [g]**		150.8 ± 61.6 (*n* = 138)	179.4 ± 73.6 (*n* = 61)	128.2 ± 37.3 (*n* = 77)	**<0.001**
**Diastolic mass/BSA [g/m^2^]**		82.3 ± 30.8 (*n* = 135)	95.2 ± 36.3 (*n* = 60)	71.9 ± 20.7 (*n* = 75)	**<0.001**
**LGE in MRI [*n* (%)]**		53 (31%) (*n* = 137)	31 (37%) (*n* = 61)	22 (26%) (*n* = 76)	**0**.**013**
**Cardiac device therapy at baseline**					
**Device already implanted at baseline [*n* (%)]**		12 (7%)	10 (12%)	2 (2%)	**0**.**032**
	*ICD* [*n* (%)]	6 (4%)	4 (5%)	2 (2%)	**0**.**024**
	*Pacemaker* [*n* (%)]	6 (4%)	6 (7%)	0 (0%)	
**Reason for implantation of a cardiac device**					
	*AV-block* [*n* (%)]	3 (2%)	3 (4%)	0 (0%)	**0**.**029**
	*Symptomatic bradycardia* [*n* (%)]	3 (2%)	3 (4%)	0 (0%)	
	*VT* [*n* (%)]	5 (3%)	4 (5%)	1 (1%)	
	*Prophylaxis* [*n* (%)]	1 (1%)	0 (0%)	1 (1%)	
**Median follow-up times for subgroup analysis**					
**Development of LGE [years]**			3.2		
**Cardiac device implantation [years]**			5.1		

Baseline characteristics are presented for all included patients, stratified by median IENFD (cutoff: > 3.8 fibers/mm). Variables were expressed as mean ± standard deviation (SD). Categorical variables are shown as count and percentage. Group differences were assessed as described in the methods section.

Abbreviations: AV = atrioventricular; BL = baseline; BSA = Body surface area; ENT = earth, nose, throth; GFR (MDRD) = glomerular filtration rate (modification of diet in renal disease); GI = gastro-intestinal; IENFD = intraepidermal nerve fiber density; IVSd = interventricular septal thickness at diastole; KDIGO = Kidney Disease: Improving Global Outcomes; LGE = late gadolinium enhancement; LVPWd = left ventricular posterior wall at diastole; MRI = magnetic resonance imaging; PNS = peripheral nervous system; UACR = Urine albumin-creatinine ratio; VT = ventricular tachycardia.

FD-specific laboratory parameters showed lower enzyme activity (0.11 ± 0.13 nmol/min/mg protein vs. 0.23 ± 0.15 nmol/min/mg protein, *P* < 0.001) and higher lyso-Gb3 values (46.8 ± 57.3 ng/mL vs. 17.9 ± 33.7 ng/mL. *P* < 0.001) in patients with reduced IENFD. Renal function was more severe reduced (GFR: 73.6 ± 28.3 mL/min/1.73 m^2^ vs. 100.3 ± 25.4 mL/min/1.73 m^2^) and cardiac markers were measured higher (hsTroponin: 49.2 ± 91 pg/mL vs. 10.6 ± 12.4 pg/mL, *P* < 0.001; NTproBNP: 822.9 ± 1207.6 pg/mL vs. 267.9 ± 586.6 pg/mL, *P* < 0.001).

Variant-specific comparisons between the late-onset *p.N215S* variant and classical FD variants are presented in the supplementary appendix (Supplementary material online, *Table S3S*). Patients carrying the late-onset variant were significantly older at baseline (41 ± 14 vs. 51 ± 16, *P* < 0.001) and showed no differences in the extent of cardiac involvement compared with those with classical variants (*P* = 0.354). However, they exhibited less frequent renal (*P* = 0.006), cerebrovascular (*P* = 0.003), gastrointestinal (*P* < 0.001), and ENT (*P* = 0.004) involvement. Cornea verticillata (*P* < 0.001) and angiokeratomas (*P* < 0.001) were also observed less often.

Neurological manifestations (particularly FD-related pain) were significantly less prevalent in the late-onset group, and CDT assessed by QST were better preserved. IENFD did not differ between groups. In summary, although carriers of the *p.N215S* late-onset variant displayed a milder neurological phenotype compared with classical FD, cardiac involvement was comparable, and no differences were observed in IENFD at the distal leg (*P* = 0.779).

### Relation between IENFD and age

IENFD was lower in male patients compared to female patients. *Figure 3B* shows IENFD correlated to age stratified by sex. IENFD decreases over lifetime. Male patients show a lower IENFD at any age compared to female patients. The youngest (male) patient with a complete loss of IENFs was 39 years old. A complete loss of IENF was not seen in female patients. Linear regression model showed the rate of decrease was similar in male and female patients over all age groups (male: IENFD = 7.34–0.1*Age, *P* < 0.001, *R*^2^ = 0.27; female: IENFD = 11.08–0.11*Age, *P* < 0.001, *R*^2^ = 0.23). Effect size measurement showed strong effects for both sexes (Cohens *d*—male: 0.61; female: 0.55).

### Defining IENFD cut-off values for outcome prediction

**Figure 4:**
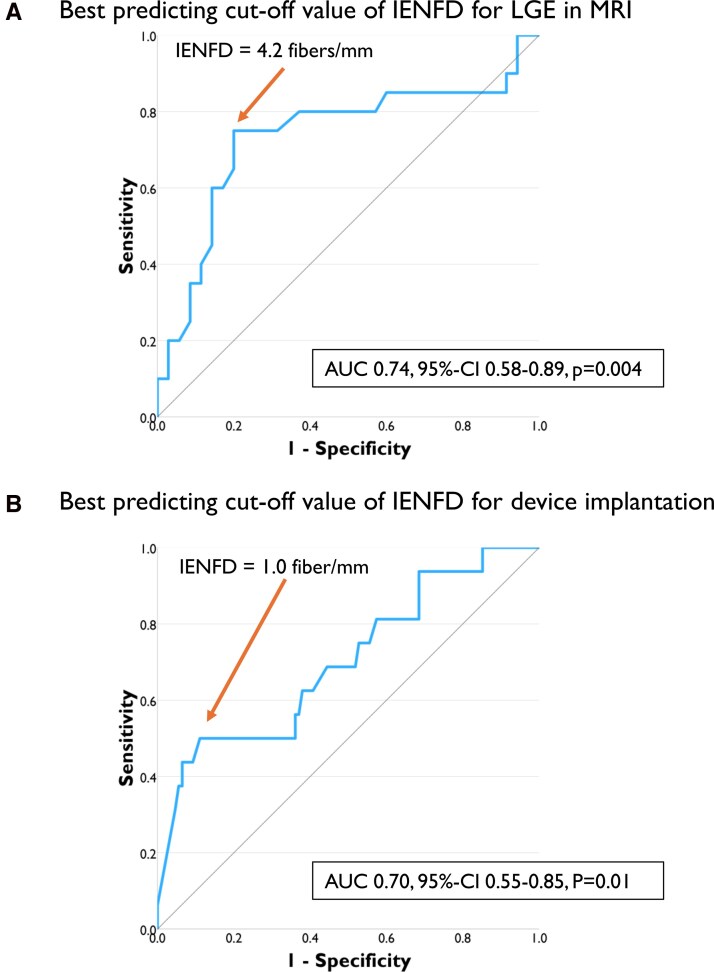
Prediction of IENFD cut-off values for both endpoints. ROC curves were used to determine the optimal cut-off values for the predefined endpoints. Using the Youden Index derived from the ROC analysis, an IENFD threshold of 4.2 fibers/mm was identified as optimal for predicting LGE in cardiac MRI, while an IENFD threshold of 1 fiber/mm was optimal for predicting cardiac device implantation. Abbreviations: **AUC** = area under the curve; **IENFD** = intraepidermal nerve fiber density; **LGE** = late gadolinium enhancement; **ROC** = receiver operating characteristic.

We examined IENFD as a potential predictive parameter for two cardiac endpoints associated with poor clinical outcome. Patients were stratified for IENFD. ROC curve analysis, shown in *Figure 4*, revealed that an IENFD of 4.2 fibers/mm best predicted LGE in cardiac MRI (AUC: 0.74, 95% CI: 0.58–0.89, *P* = 0.004) and an IENFD of 1.0 fiber/mm the implantation of cardiac devices (AUC: 0.7, 95% CI: 0.55–0.85, *P* = 0.01).

### Event free time: LGE in cardiac MRI

**Figure 5:**
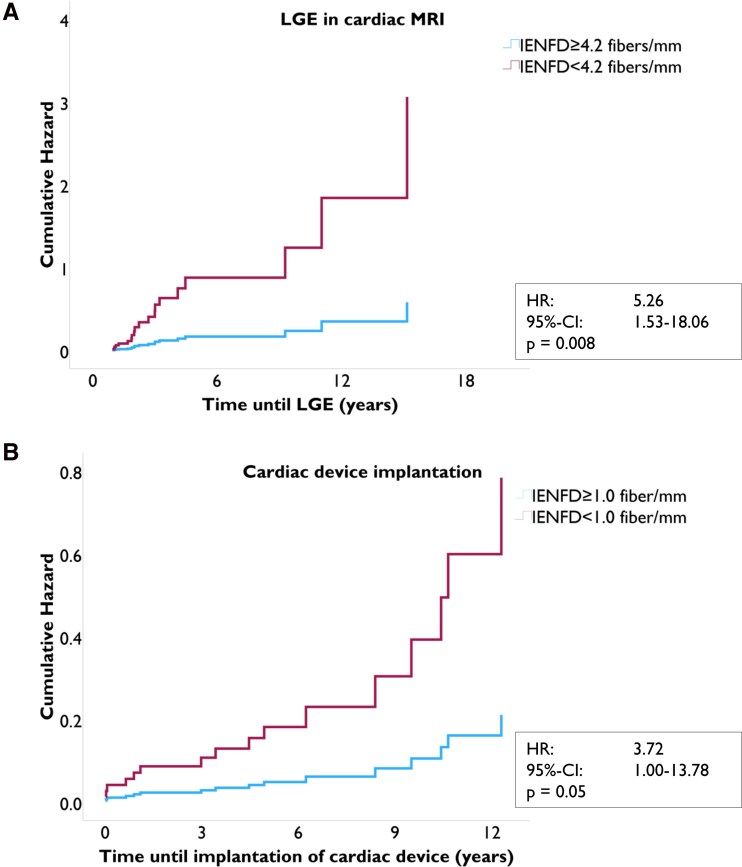
Cox regression models for the two cardiac endpoints. Figure 5 depicts the multivariable Cox regression models illustrating IENFD as a prognostic marker for the development of LGE (panel A) and for cardiac device implantation (panel B). Results are presented as hazard functions over time (in years). IENFD was confirmed as an independent predictor for both endpoints. Both models reached statistical significance and validated IENFD as a prognostic indicator. Abbreviations: **CI** = confidence interval; **IENFD** = intraepidermal nerve fiber density; **LGE** = late gadolinium enhancement.

Fifty-five patients were included in this first subgroup analysis (*Figure 1*). Thirty-three patients had an IENFD ≥ 4.2 fibers/mm, while 22 patients had < 4.2 fibers/mm. The median follow-up time for this endpoint was 3.2 years. Univariable Cox-regression model identified age (HR: 1.35, 95% CI: 1.06–1.72, *P* = 0.016) and IENFD < 4.2 fibers/mm (HR: 9.26, 95% CI: 3.04 vs. 28.25, *P* < 0.001) as risk factors for the development of LGE. Sex was not associated with the outcome (*P* = 0.108). Combining these results in one multivariable Cox-regression analysis adjusted for age and sex, IENFD < 4.2 remained as an independent risk factor for developing LGE in cardiac MRI with a 5.2 times higher risk (HR: 5.26, 95% CI: 1.53–18.06, *P* = 0.008) as shown in *Figure 5A* and Supplementary material online, *Table S6S*.

### Excluded patients (LGE in cardiac MRI)

Fifty-three patients already had LGE at baseline. Additional 33 patients had to be excluded because of missing MRI data. Reasons for exclusion of these patients are shown detailed in Supplementary material online, *Table S1S* (see supplementary appendix). Patients were excluded because of already implanted pacemaker or ICD (*n* = 12, 36%), severe renal dysfunction (GFR < 35 mL/min/1.73^2^) (*n* = 6, 18%) or other, non-FD-related, reasons (claustrophobia, contrast agent allergy or technical artefacts). In patients who were excluded because of an implanted cardiac device, IENFD was reduced with 1.2 [0.2–2.7] fibers/mm. This was also seen in patients with severe renal impairment (IENFD mean: 1 ± 0.8 fibers/mm).

### Event free time: cardiac device therapy

The second endpoint was defined as ICD or pacemaker implantation. One hundred and twenty-four patients were included with a median follow-up time of 5.1 years (*Figure 1*). Again ROC-curve analysis was used to determine the most predictive cut-off value, which was an IENFD < 1 fiber/mm (*Figure 4B*). Univariable Cox-regression model also identified age (HR: 1.35, 95% CI: 1.09–1.67, *P* = 0.006) and an IENFD < 1 fiber/mm (HR: 5.88, 95% CI: 2.17–15.92, *P* < 0.001) as risk factors for the need of a device therapy. Sex had no impact on the outcome (*P* = 0.143). Combining all factors in one multivariable Cox-regression analysis, IENFD < 1 fiber/mm adjusted for sex and age remained an independent risk factor (HR: 3.72, 95% CI: 1.00–13.78, *P* = 0.05) (compare *Figure 5B* and Supplementary material online, *Table S6S*).

### Reasons for pacemaker or ICD implantation

In Supplementary material online, *Table S2S* reasons for pacemaker or ICD implantation are shown. Seventeen devices were implanted over the follow-up period, with 12 (71%) ICD devices and 5 (29%) pacemakers. Recorded or symptomatic VTs were the main reason for ICD implantation [9 (75%)]. Three (25%) devices were implanted prophylactically given the higher risk for sudden cardiac death in FD.^43^ Reasons for pacemaker implantation were symptomatic sinus bradycardia in 4 (80%) cases and in one (20%) case higher-grad AV-blocking. Baseline IENFD in patients with recorded VT was 0.5 [0.1–2.5], in symptomatic sinus bradycardia 1.9 [0.1–4.2]. Patients with prophylactic ICD-implantation had an IENFD of 6.6 [3.3–7.9].

### Model evaluation for enhancing predictive accuracy

Significant differences in the CDT derived from QST testing were observed between both groups. Consequently, QST was tested as a covariate in both endpoint models. In the model predicting the development of LGE, QST showed an association with prediction; however, nested model comparisons indicated that IENFD was a more informative parameter. Regarding the prediction of device implantation, QST was not significantly associated. Furthermore, nested models demonstrated that the initial model excluding QST exhibited greater robustness. Therefore, we elected to exclude the QST CDT as a covariate from the primary model. A detailed rationale is provided in the supplementary appendix.

## Discussion

This study analysed long-term data from FD patients who underwent skin punch biopsy for the assessment of IENFD. It is well established in literature that skin innervation declines physiologically with age, and that IENFD is typically lower in men compared to women.^44,45^ Consistent with these findings, our FD cohort demonstrated lower IENFD values in men relative to women, as well as reduced IENFD in older patients compared to younger individuals. A complete loss of epidermal innervation was almost exclusively seen in men. The decrease of IENFD was similar in both male and female patients (compare linear regression in *Figure 3B*) and were similar to previously published data.^12^ Further, a strong negative correlation between IENFD and measured GFR has been described.^9,12^ In our current work, we analysed if and how IENFD and FD cardiomyopathy are connected. Our approach was to evaluate relevant cardiac endpoints (LGE in cardiac MRI and device implantation) for their prognostic value.

As shown in the baseline characteristics, the presence of peripheral nervous system symptoms cannot be explained by FD associated small fiber pathology only in terms of a reduction in IENFD. While this may seem paradoxical, similar results had previously been found in a small study. In this mentioned research, a differentiation between FD patients and healthy individuals using IENFD in skin punch biopsies was not possible and reduced IENFD did not correlate with FD pain.^13^ Hence, mere investigation of skin innervation is not suitable to determine the presence of a relevant peripheral nervous system impairment or to diagnose FD. It is, however, intriguing, that patients with a pronounced cardiac phenotype or kidney dysfunction often had a greatly reduced IENFD.

Baseline characteristics showed that a distinct cardiac phenotype was more often seen in patients with reduced IENFD. While IENFD physiologically depends on age and sex, our analysis revealed no such interrelation when assessing data in highly pathological ranges. Severe deterioration of IENFD was seen much earlier, typical for FD. Nevertheless, age and sex remained possible confounding variables in our analysed cohort.

Further certainty was achieved through the analysis of longitudinal data. IENFD was identified as an independent predictor of adverse cardiac events in FD, as reflected by the development of LGE on cardiac MRI or the requirement for cardiac device implantation (*Figure 5*). Cox regression models were adjusted for age and sex, and IENFD remained an independent prognostic factor in the multivariable analysis for both cardiac endpoints.

LGE on cardiac MRI is indicative of myocardial fibrosis, likely resulting from the deleterious effects of lysosomal storage product accumulation and associated disruptions in cellular metabolism.^46,47^ For LGE measurement in cardiac MRI, some prerequisites are obligatory. Especially, implanted cardiac devices or severe kidney dysfunctions are relative contraindications for MRI examination or applying contrast agents. However, these patients are the most severely affected FD patients. Exclusion of those could be confounding the results. In Supplementary material online, *Table S1S* (see supplementary appendix) IENFD values of these patients are shown (excluded because of cardiac device already implanted (*n* = 12): IENFD: 1.2 [0.2–2.7]; excluded because of severe renal impairment (*n* = 6): IENFD: 1.0 ± 0.8). These severely affected FD patients, who were excluded also showed a strong reduction of IENFD, which may support our findings.

We suggest that pathological processes in FD may be detectable early in the disease course via IENFD assessment, as they occur simultaneously in other affected organ systems, such as the heart, where they often remain unrecognized at this disease stage. The natural course of FD with typically burning acral pain in early childhood, impaired temperature perception and/or dyshidrosis as first symptoms underscore our hypothesis.^5^ These symptoms are assigned to the small calibre nerve fibers. We assume, that early damage in peripheral nerve fibers may also predict a high disease activity in other cells like cardiomyocytes.

The second endpoint was the implantation of a cardiac device. Interestingly, best predicting cut-off value was < 1 fiber/mm. This value is very low and as shown in *Figure 3B* was almost only seen in male patients. Upon closer inspection why devices were implanted, differences were found in IENFD, as shown in Supplementary material online, *Table S2S* (see supplementary appendix). In patients with symptomatic VT or bradycardia, IENFD was strongly reduced [bradycardia (*n* = 4): IENFD: 1.9 (0.1–4.2); VT (*n* = 9): IENFD: 0.5 (0.1–2.5)]. Patients receiving an ICD-therapy for primary prophylaxis had a higher IENFD [prophylaxis (*n* = 3) IENFD: 6.6 (3.3–7.9)]. Notably, patients receiving a cardiac device for secondary indications exhibited lower IENFD than those undergoing implantation for primary prophylaxis, suggesting that primary prophylactic device implantation may have occurred at an earlier (possibly premature) stage of disease progression. In our analysis, we used a combined endpoint encompassing both bradycardic and tachycardic rhythm disorders, defined by the need for cardiac device implantation. Although this approach may appear counterintuitive, as these rhythm disorders may arise from different underlying pathophysiological mechanisms, prior evidence supports this strategy. In a study employing implantable loop recorders in FD patients, both bradycardic and tachycardic rhythm disorders were detected and prompted changes in clinical management, including cardiac device implantation.^48^ On this basis, both bradycardic and tachycardic rhythm disorders were regarded as manifestations of disease progression in FD and were analysed as a unified endpoint. In the Supplementary appendix, we additionally analysed ICD implantation as a separate endpoint, which identified tachycardiac events as the primary driver of the combined device endpoint (Supplementary material online, *Figure S2S*). Due to the very small number of pacemaker implantations (*n* = 5), a separate analysis of this endpoint was not feasible or clinically meaningful. Nevertheless, both event types carry relevant therapeutic consequences, and IENFD demonstrated prognostic information across the combined endpoint.

A huge challenge in FD diagnostics is the prediction of individual disease courses and therapeutic decisions in early disease stages.^49^ Especially in women, mildly affected male patients or patients carrying late-onset variants it remains unclear how FD will develop over time. As seen in the SOPHIA study, which examined cardiac features of FD exclusively in women, LGE in terms of cardiac fibrosis, can occur earlier than LVH.^35^ Risk stratification with IENFD may be an easily accessible option in these unclear cases to better predict the cardiac disease course. Furthermore, IENFD may be suitable as an additional tool for making therapy decisions. In advanced stages of FD, correct timing of the implantation of cardiac devices remains an obstacle.^21,50^ Established scores predicting the risk for sudden cardiac death in HCM patients are not applicable in metabolic diseases like FD.^51,52^ Our analysis demonstrates that patients with reduced IENFD (< 1 fiber/mm) have a higher risk to develop severe cardiac arrhythmias leading to device implantation. Beyond established risk factors such as left ventricular hypertrophy and extensive myocardial scarring assessed by LGE on cardiac MRI, IENFD may help for therapeutic risk stratification of patients at increased risk of malignant ventricular arrhythmias.^43^ However, patients in our cohort with implanted devices for primary prevention showed higher IENFD. This raises the question of whether patients truly benefit from device therapy at this specific time point, considering the associated procedural risks and potential long-term complications such as device associated infections or thrombosis.^53^

Limitations in our work arise from the single-centre design of this study. Potential therapy effects were not addressed and evaluated in our analysis. Baseline was defined only by date of skin punch biopsy, which may also be confounding for different other factors. The endpoint of LGE in cardiac MRI was analysed as a binary variable (presence vs. absence), which may oversimplify the underlying myocardial pathological processes. Quantitative assessment of LGE could have provided greater precision and strengthened the robustness of the endpoint. However, given that data from this cohort were collected over a 16-year period, methodological consistency required for reliable quantitative analyses could not be fully ensured; therefore, such analyses were not performed. As follow-up visits were typically conducted annually in our disease programme and included cardiac MRI examinations, we considered the use of a binary LGE endpoint to be appropriate and feasible for our analysis.

Another limitation arises from the missing external validation of our IENFD cut-off values, which was not feasible due to the rarity of FD. To our knowledge, no independent external cohort currently exists with both skin punch biopsy data and cardiac MRI examinations available.

During model development, the proportional hazards assumption was formally assessed using time-dependent covariates; however, a time-varying effect for IENFD was observed, indicating that the assumption was not fully met. Cumulative hazard curves suggested that the association between low IENFD and cardiac outcomes was strongest early during follow-up and attenuated over time. Accordingly, the reported hazard ratios should be interpreted as average effects across the study period. Limited by the small number of events, more complex time-varying models were not pursued.

Taken together these limitations we support that prospective, multicentre studies with harmonized imaging protocols, systematic assessment of therapeutic exposure, and integrated cardiac outcome measurements are warranted to validate the proposed IENFD cut-off values and to clarify the optimal use of this parameter in a controlled clinical setting. A deeper understanding of interaction between different organ systems should be aimed at. Important endpoints like renal failure or cerebrovascular events as major life-shortening events in FD should also be examined.

Still, our analysis demonstrates strong evidence that IENFD includes prognostic information for the development of severe cardiac organ manifestations. We advocate that skin punch biopsy should be included in all patients for disease evaluations, not only for purposes of peripheral nervous system examination.

## Conclusion

Decrease of IENFD determined in distal skin punch biopsies of FD patients contains prognostic information about the future course of FD cardiomyopathy. Patients with low IENFD were at higher risk of developing cardiac fibrosis. Patients with severely reduced IENFD had a higher risk receiving a cardiac device therapy. Given its potential prognostic value, IENFD assessment should be incorporated into disease evaluation programmes, particularly to make external validation of our findings possible. Further prospective, multi-centre studies should confirm these results and verify, whether IENFD can also predict future nephrological or cerebrovascular complications.

## Lead author biography


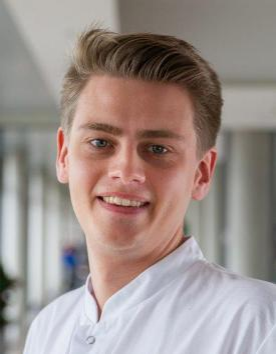
 **Kolja Lau, MD**, began his research career in 2017 at the University Hospital Würzburg, Germany, where he focused on echocardiographic parameters in heart failure as part of his doctoral thesis. He is currently an assistant physician at one of Germany’s leading Fabry disease centres, where comprehensive patient care and the longitudinal management of this specialized patient population are central to his clinical work. Since 2021, his clinical and scientific activities have been dedicated to Fabry disease, with a particular focus on variants of uncertain significance, novel therapeutic approaches, and the diagnosis and management of Fabry cardiomyopathy.

## Supplementary Material

oeag042_Supplementary_Data

## Data Availability

The data underlying this article will be shared on reasonable request to the corresponding author.
