# Supplementary material for: Intraepidermal nerve fiber density as a predictor of cardiac events in Fabry disease
Source: Eur Heart J Open. 2026 Mar 6;6(2):oeag042. doi: 10.1093/ehjopen/oeag042 (PMC13089518; doi:10.1093/ehjopen/oeag042)
Supplement: oeag042_Supplementary_Data [file oeag042_supplementary_data.zip › 3 IENFD_Supplementary_material_clean_version_revision_II.docx]

**S U P P L E M E N T A R Y A P P E N D I X**

| Reason why MRI data was missing | | n  33 (100.0 %) |
| --- | --- | --- |
| Cardiac device already implanted | | 12 (36 %) |
| Severe renal impairment | | 6 (18 %) |
| Claustrophobia | | 6 (18 %) |
| contrast agent allergy | | 1 (3 %) |
| Other reason | | 8 (24 %) |
| Parameter | **Cardiac device implanted**  **(n = 12)** | **Severe renal impairment**  **(n = 6)** |
| Age | 60 ± 6 | 48 ± 12 |
| IENFD | 1.2 [0.2-2.7] | 1 ± 0.8 |

**Table 1s:** Missing MRI data for endpoint development of LGE in MRI

Table 1s summarizes the reasons for the absence of MRI data for endpoint 1 analysis (Development of LGE). A total of 33 patients were excluded due to missing MRI data. The lower section presents age and IENFD metrics for patients excluded based on disease-specific criteria (presence of cardiac devices or severe renal impairment). Continuous variables are presented as mean ± SD if normally distributed, and as median [IQR] if non-normally distributed.

Abbreviations: **IENFD** = intraepidermal nerve fiber density; **LGE** = late gandolinium enhancement; **MRI** = magnetic resonance imaging.

|  | Reasons for implantation of cardiac devices over Follow-up period (n = 17) | | | | | | |
| --- | --- | --- | --- | --- | --- | --- | --- |
| Device: | | n (%) | Symptomatic bradycardia | AV-Block | | Ventricular tachycardia | Prophylaxis |
| Pacemaker | | 5 (29 %) | 4 (80 %) | 1 (20 %) | |  |  |
| ICD | | 12 (71 %) |  |  | | 9 (75 %) | 3 (25 %) |
|  | **Reasons for implantation and IENFD values (mean ± standard deviation)** | | | | | | |
| Reasons for implantation | | | | | IENFD | | |
| Bradycardia (n = 4) | | | | | 1.9 [0.1-4.2] | | |
| Ventricular tachycardia (n = 9) | | | | | 0.5 [0.1-2.5] | | |
| AV-Block (n = 1) | | | | | 6.6 | | |
| Prophylaxis (n =3) | | | | | 6.6 [3.3-7.9] | | |

**Table 2s:** Reasons for implantation of cardiac devices over Follow-up period

Table 2s details cardiac device implantations during the follow-up period, including associated indications. A total of 17 devices were implanted: 5 pacemakers and 12 ICDs. Symptomatic bradycardia was the primary indication for pacemaker therapy, while symptomatic ventricular tachycardia prompted ICD therapy. Due to small sample size, continuous variables are presented as median [IQR].

Abbreviations: **AV** = atrioventricular; **ICD** = Implantable cardioverter defibrillator **IENFD** = intraepidermal nerve fiber density.

| Parameter | Classic variant  (n=129) | Late onset  (p. N215S)  (n=41) | p |
| --- | --- | --- | --- |
| Male sex [n (%)] | 59 (46%) | 21 (51%) | 0.592 |
| Age [years] | 41 ± 14 | 51 ± 16 | <.001 |
| Organ involvement | | | |
| Heart [n (%)]  (any signs of disease) | 84 (65%) | 23 (56%) | 0.354 |
| Kidney [n (%)]  (KDIGO stage G1A2 and lower) | 56 (43%) | 8 (20%) | **0.006** |
| Cerebrovascular [n (%)]  (TIA or stroke) | 28 (22%) | 1 (2%) | **0.003** |
| Any symptoms of peripheral nervous system [n (%)] | 91 (71%) | 14 (34%) | **0.001** |
| *Hyperhidrosis* [n (%)] | 11 (8.5%) | 9 (22%) | **0.027** |
| *Hypo-/anhidrosis* [n (%)] | 56 (43%) | 4 (10%) | **<.001** |
| *FD pain* [n (%)] | 82 (64%) | 9 (22%) | **<.001** |
| QST cold detection threshold (value) | -8.6 ± 7.6  (n=128) | -4.6 ± 4.5 | **0.008** |
| QST cold detection threshold (z-score) | -0.8 ± 1.3  (n=128) | -0.2 ± 0.9 | **0.005** |
| GI- symptoms [n (%)]  (any reported symptoms) | 54 (42%) | 5 (12%) | **<.001** |
| Cornea verticillata [n (%)]  (ophthalmologic diagnosed) | 54 (42%) | 0 (0%) | **<.001** |
| ENT [n (%)]  (any reported symptoms) | 78 (61%) | 14 (34%) | **0.004** |
| Angiokeratoma [n (%)] | 48 (37%) | 1 (2%) | **<.001** |
| Ongoing FD specific therapy or indication seen at BL [n (%)] |  |  |  |
| *FD therapy at baseline* [n (%)] | 32 (25%) | 8 (20%) |  |
| *FD therapy indication seen at*  *baseline* [n (%)] | 60 (47%) | 14 (34%) |  |
| Skin biopsy | | | |
| Reduced IENFD reported [n (%)] | 90 (70%) | 29 (71%) | 1.0 |
| IENFD distal leg [fibers/mm] | 4.7 ± 3.8 | 4.2 ± 2.9 | 0.779 |
| IENFD back [fibers/mm] | 20.4 ± 9.6 | 21.2 ± 7.7 | 0.597 |
| Cardiac/Kidney/FD parameters | | | |
| IVSd - echocardiography [mm] | 11 ± 2.9 | 11.4 ± 4 | 0.898 |
| LVPWd - echocardiography [mm] | 10.5 ± 2.4 | 10.4 ± 3.5 | 0.281 |
| A-Gal enzyme activity in leucocytes  [nmol/min/mg protein] | 0.16 ± 0.14  (n=121) | 0.2 ± 0.17  (n=39) | **0.038** |
| Lyso-Gb3 [ng/ml] | 42.3 ± 54  (n=116) | 5.2 ± 4.8 | **<.001** |
| GFR (MDRD) [ml/min/ 1.73 qm] | 86.9 ± 32.5  (n=127) | 87.9 ± 20.3 | 0.795 |
| Cystatin C [mg/l] | - 1. ± 0.9   (n=122) | 0.9 ± 0.2 | 0.496 |
| UACR [mg/g creatinine] | 257.3 ± 631.5  (n=121) | 146.8 ± 447.7 | 0.404 |
| Troponin T (hs) [pg/ml] | 30.4 ± 69.5  (n=55) | 34.2 ±73  (n=34) | 0.423 |
| NT-proBNP [pg/ml] | 488.8 ± 979.1  (n=122) | 666.3 ± 958.6 | 0.275 |
| MRI parameters | | | |
| Data available [n (%)] | 104 (81%) | 33 (81%) | 1.0 |
| Diastolic mass [g] | 149 ± 54.9  (n=104) | 157 ± 80  (n=33) | 0.533 |
| Diastolic mass/ BSA [g/m^2^] | 81.8 ± 28  (n=102) | 84 ± 39  (n=33) | 0.357 |
| LGE in MRI [n (%)] | 39 (30%)  (n=104) | 14 (34%)  (n=33) | 0.683 |

**Table 3s:** Comparison between late onset variant (p.N215S) and other FD variants

Baseline characteristics are presented splitted by genetic variants. All classic variants are shown in comparison to late onset variant (p.N215S). Variables were expressed as mean ± standard deviation (SD). Categorical variables are shown as count and percentage. Group differences were assessed as described in the methods section.

| **Genetic variant (GLA-gene)** | **Count (n)** | **Percantage (%)** |
| --- | --- | --- |
| 42del TGCGCTT + -10C>T, IVS4-16A>G | 1 | 0.6 |
| c.-110-15T>G | 1 | 0.6 |
| c.1091_1092 CT Deletion | 1 | 0.6 |
| c.162del T | 1 | 0.6 |
| c.756 or 757 del A, fs 268X | 3 | 1.8 |
| c.993_994 ins A (fs X 338) | 6 | 3.5 |
| Deletion c.863delC // p.Ala288Valfs*29 | 1 | 0.6 |
| Deletion c.1029_1030 del TC fs X30 | 1 | 0.6 |
| Deletion c.1062_1076del, I354fsdel 15bp | 1 | 0.6 |
| Deletion c.1221 del A Ile407fsX10 | 1 | 0.6 |
| Deletion c.1221 del A (Ile407fsX10) | 1 | 0.6 |
| Deletion c.1223 del A (p.Asn408IlefsX10) | 2 | 1.2 |
| Deletion c.718-719 del AA (fs 248X) | 1 | 0.6 |
| Deletion c.972del G | 2 | 1.2 |
| Deletion of exon 2 | 1 | 0.6 |
| Deletion of exon 3 | 1 | 0.6 |
| Intron 2, IVS2+1 (G>A) | 2 | 1.2 |
| Intron 3, IVS3+1 G>A | 1 | 0.6 |
| Intron 6, Transition IVS6-10G>A | 2 | 1.2 |
| Intron 6, Transition IVS6-10G>A (Splice -Site-Mutation c. 1000-10G>A) | 3 | 1.8 |
| IVS5-3_2del CA | 1 | 0.6 |
| Mutation c.1208 del AAG | 2 | 1.2 |
| p.A135V | 6 | 3.5 |
| p.A190Pfs*1 | 2 | 1.2 |
| p.A292P | 1 | 0.6 |
| p.C142Ter | 1 | 0.6 |
| p.C172Y | 3 | 1.8 |
| p.C52S | 2 | 1.2 |
| p.C63Y | 1 | 0.6 |
| p.D136E | 3 | 1.8 |
| p.D165V | 1 | 0.6 |
| p.D170N | 2 | 1.2 |
| p.E341K | 1 | 0.6 |
| p.E358del | 2 | 1.2 |
| p.G132E | 1 | 0.6 |
| p.G325S | 6 | 3.5 |
| p.G375V | 1 | 0.6 |
| p.G395A | 2 | 1.2 |
| p.H46R | 1 | 0.6 |
| p.I117S | 1 | 0.6 |
| p.K240N | 1 | 0.6 |
| p.L129P | 3 | 1.8 |
| p.L311V | 1 | 0.6 |
| p.L415R | 1 | 0.6 |
| p.L417P | 1 | 0.6 |
| p.L89F | 1 | 0.6 |
| p.M187V | 1 | 0.6 |
| p.M42V | 2 | 1.2 |
| p.N139S | 1 | 0.6 |
| p.N139S + p.W236C | 1 | 0.6 |
| p.N215S | 41 | 24.1 |
| p.N224S | 2 | 1.2 |
| p.P40L | 1 | 0.6 |
| p.Q280X | 1 | 0.6 |
| p.Q312X | 1 | 0.6 |
| p.Q357X | 3 | 1.8 |
| p.R112C | 1 | 0.6 |
| p.R112H | 2 | 1.2 |
| p.R118C | 1 | 0.6 |
| p.R227X | 3 | 1.8 |
| p.R301Q | 2 | 1.2 |
| p.R342L | 4 | 2.4 |
| p.R356Q | 1 | 0.6 |
| p.T282I | 2 | 1.2 |
| p.V269G | 2 | 1.2 |
| p.V334A | 1 | 0.6 |
| p.W162C | 1 | 0.6 |
| p.W204C | 1 | 0.6 |
| p.W204X | 2 | 1.2 |
| p.W236C | 3 | 1.8 |
| p.W262R | 1 | 0.6 |
| p.W287S | 1 | 0.6 |
| p.W349X | 2 | 1.2 |
| p.W399S | 1 | 0.6 |
| p.W399X | 2 | 1.2 |
| p.Y216X | 2 | 1.2 |
| **Overall:** | **170** | **100** |

**Table 4s:** Genetic variants of AGAL-gene

Table 4s presents the spectrum of genetic variants identified in the alpha-galactosidase A enzyme (GLA gene) within the patient cohort. Amino acid changes are reported using standard single-letter codes, while genomic nucleotide sequences are provided for intronic variants and deletions.


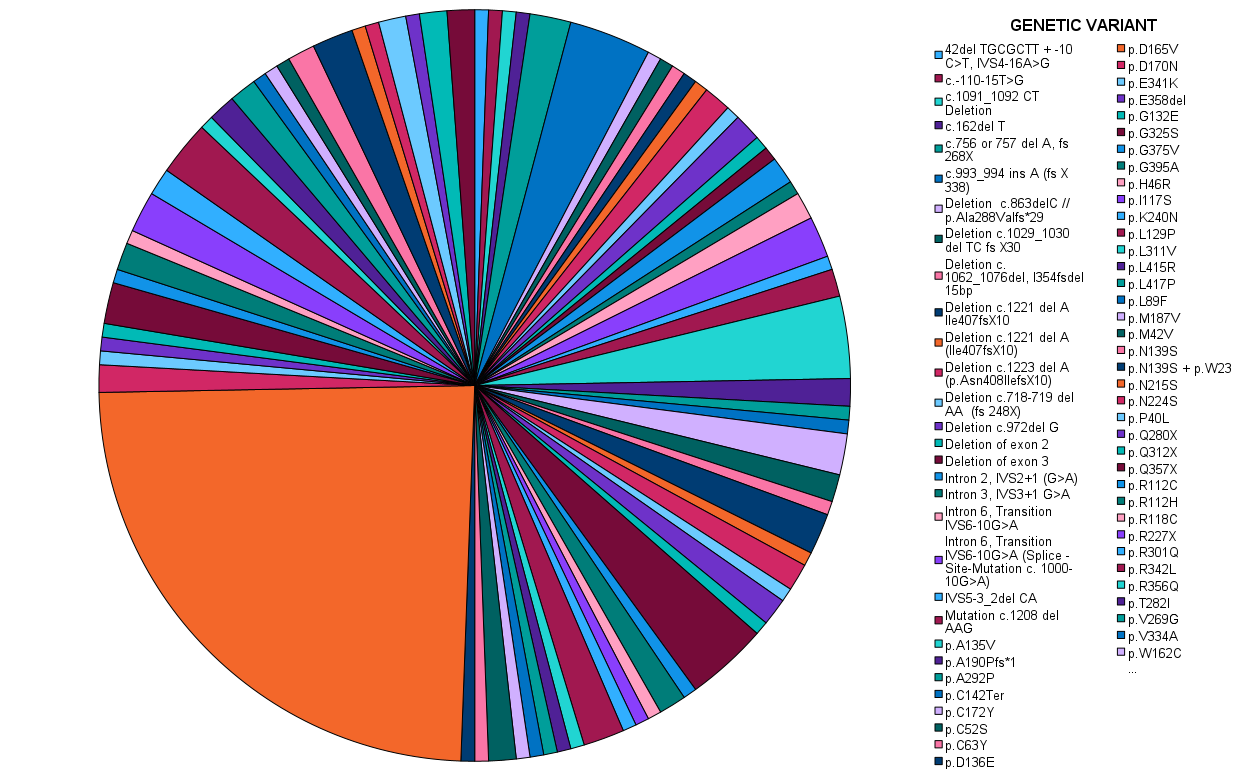


p.N215S

n=41

**Figure 1s:** Distribution of patients stratified by genetic variants

Figure 1s illustrates the distribution of the identified genetic variants. Notably, the p.N215S variant constitutes a substantial proportion of the cohort and is classically associated with a severe cardiac phenotype.

**A D D I T I O N A L S T A T I S T I C A L A N A L Y S I S**

As detailed in the primary manuscript, we conducted an in-depth analysis of our optimal predictive model for the two outcome variables: late gadolinium enhancement (LGE) on cardiac MRI and device implantation. Table 6s presents the primary used model not using results from quantitative sensory testing (QST). Intraepidermal nerve fiber density (IENFD) emerged as a significant predictor for both endpoints. Given that baseline characteristics demonstrated statistically significant differences in QST cold detection thresholds between groups stratified by IENFD, cold detection threshold was subsequently tested as a relevant covariate in the Cox proportional hazards regression analysis.

The initial step in model refinement involved evaluating collinearity between IENFD and QST parameters. Collinearity was assessed through Spearman’s rank correlation coefficient and variance inflation factors (VIFs) derived from multivariable Cox regression models, with a predefined VIF threshold of <2 indicating negligible collinearity. Both variables were retained in the model when collinearity was deemed non-significant. Specifically, QST cold detection threshold exhibited a moderate correlation with IENFD measured at the lower leg (Spearman’s ρ=0.417, p<.001), suggesting these metrics capture related yet distinct facets of small-fiber dysfunction. The low level of collinearity (approximate VIF=1.21) further justified the concurrent inclusion of both variables in the multivariable Cox models (Table 5s).

|  | | | **QST cold detection threshold (value)** | **IENFD** |
| --- | --- | --- | --- | --- |
| Spearman's rho | QST cold detection threshold (value) | Correlation Coefficient | 1.000 | .417^**^ |
|  |  | Significance (2-tailed) | . | **<.001** |
|  |  | n | 169 | 169 |
|  | IENFD | Correlation Coefficient | .417^**^ | 1.000 |
|  |  | Significance (2-tailed) | **<.001** | . |
|  |  | n | 169 | 170 |
| **. Correlation is significant at the 0.01 level (2-tailed). | | | | |

**Table 5s:** Spearman correlation between IENFD and QST cold detection threshold

Table 5s shows the spearman correlation between IENFD and QST cold detection threshold indicating a correlation between the two variables.

Abbreviations: **IENFD** = intraepidermal nerve fiber density; **QST** = quantitative sensory testing.

| **Cox regression models for predicting LGE development in FD patients** | | | |  | **Cox regression models for predicting device implantation** | | | |
| --- | --- | --- | --- | --- | --- | --- | --- | --- |
| Univariable |  | HR (95%-CI) | P value |  | Univariable |  | HR (95%-CI) | P value |
| Age (per 5 years) |  | 1.35 (1.06-1.72) | 0.016 |  | Age (per 5 years) |  | 1.35 (1.09-1.67) | 0.006 |
| Male vs. Female |  | 2.10 (0.85-5.20) | 0.108 |  | Male vs. Female |  | 2.14 (0.77-5.94) | 0.143 |
| IENFD (fibers/mm) |  | 0.75 (0.63-0.88) | <0.001 |  | IENFD (fibers/mm) |  | 0.79 (0.66-0.95) | 0.014 |
| IENFD ≥4.2 | 5/33 (15.2%) | reference |  |  | IENFD ≥1.0 | 9/107 (8.4%) | reference |  |
| IENFD <4.2 | 15/22 (68.2%) | 9.26 (3.04-28.25) | <0.001 |  | IENFD <1.0 | 7/17 (41.2%) | 5.88 (2.17-15.92) | <0.001 |
| P value | <0.001 |  |  |  | P value | 0.001 |  |  |
|  |  |  |  |  |  |  |  |  |
| Multivariable |  | HR (95%-CI) | P value |  | Multivariable |  | HR (95%-CI) | P value |
| Age (per 5 years) |  | 1.80 (1.22-2.64) | 0.003 |  | Age (per 5 years) |  | 1.38 (1.09-1.74) | 0.007 |
| Male vs. Female |  | 6.50 (1.44-29.12) | 0.015 |  | Male vs. Female |  | 1.64 (0.41-6.57) | 0.485 |
| IENFD ≥4.2 |  | reference |  |  | IENFD ≥1.0 |  | reference |  |
| IENFD <4.2 |  | 5.26 (1.53-18.06) | 0.008 |  | IENFD <1.0 |  | 3.72 (1.00-13.78) | 0.050 |
|  |  |  |  |  |  |  |  |  |
| *Age, sex, and IENFD<4.2 were entered as covariates into the multivariable Cox regression model using the "Enter" method.* | | | |  | *Age, sex, and IENFD<1.0 were entered as covariates into the multivariable Cox regression model using the "Enter" method.* | | | |

**Table 6s:** *Table 6s shows the main Cox regression models predicting LGE and device implantation.*

Abbreviations: **CI** = confidence interval; **HR** = hazard ratio; **IENFD** = intraepidermal nerve fiber density; **LGE** = late gadolinium enhancement; **MRI** = magnetic resonance imaging.

In the next step we added QST testing into the established model as shown in Table 7s.

Event free time: LGE in cardiac MRI:

Fifty-five patients were included in this subgroup analysis (see Figure 1); 33 patients had an IENFD≥4.2 fibers/mm and 22 patients had<4.2 fibers/mm. In univariable Cox models, age (HR per 5 years: 1.35, 95%-CI 1.06–1.72, p=0.016), QST cold detection threshold (HR per unit: 0.89, 95%-CI 0.83–0.95, p<0.001), and IENFD<4.2 (HR: 9.26, 95%-CI 3.04–28.25, p<0.001) were associated with the development of LGE; sex was not (p=0.108). In the multivariable enter model adjusted for age, sex, and QST cold detection threshold (value), IENFD<4.2 remained independently associated with incident LGE (HR: 6.20, 95%-CI 1.73–22.24, p=0.005), QST also remained significant (HR per unit: 0.90, 95%-CI 0.82–1.00, p=0.046), and age was retained (HR per 5 years: 1.54, 95%-CI 1.02–2.33, p=0.041); sex was not (p=0.325) (Table 7s).

Event free time: cardiac device therapy:

The second endpoint was defined as ICD or pacemaker implantation. 124 patients were included (compare Figure 1); of these, 123 had complete covariates and entered the Cox analyses (events=16). In univariable models, age (HR per 5 years: 1.35, 95%-CI 1.09–1.67, p=0.006) and IENFD<1.0 fiber/mm (HR: 5.88, 95%-CI 2.17–15.92, p<.001) were associated with device implantation, whereas QST cold detection showed a non-significant trend (p=0.062) and sex was not associated (p=0.143). In the multivariable enter model including age, sex, QST, and IENFD<1.0, age remained significant (HR per 5 years: 1.38, 95%-CI 1.09–1.74, p=0.008), QST was not associated (p=0.985), and IENFD<1.0 showed a three- to fourfold higher hazard that did not reach conventional significance (HR: 3.74, 95%-CI 0.83–16.84, p=0.085) (Table 7s).

The results prompt several key questions:

1. Does quantitative sensory testing (QST) serve as a superior predictor of late gadolinium enhancement (LGE) on cardiac MRI compared to intraepidermal nerve fiber density (IENFD)?
2. To what extent does QST influence the prediction of cardiac device implantation?

To address these questions and assess the incremental prognostic value of IENFD versus QST cold detection, we fitted nested block-entry Cox models in both directions: (Base A) age + sex + QST, then adding IENFD; and (Base B) age + sex + IENFD, then adding QST. Improvement in fit was quantified by the change in −2 log-likelihood (Δ−2LL) with 1 degree of freedom, and model parsimony was evaluated using AICc (small-sample corrected Akaike information criterion) and BIC (Bayesian information criterion) derived from the model −2LL and the number of parameters (Table 8s and Table 9s).

| **Cox regression models for predicting LGE development in FD patients** | | | |  | **Cox regression models for predicting device implantation** | | | |
| --- | --- | --- | --- | --- | --- | --- | --- | --- |
| Univariable |  | HR (95%-CI) | P value |  | Univariable |  | HR (95%-CI) | P value |
| Age (per 5 years) |  | 1.35 (1.06-1.72) | 0.016 |  | Age (per 5 years) |  | 1.349 (1.10-1.67) | 0.006 |
| Male vs. Female |  | 2.10 (0.85-5.20) | 0.108 |  | Male vs. Female |  | 2.14 (0.77-5.94) | 0.143 |
| QST cold detection threshold (value) |  | 0.89 (0.83-1.00) | <0.001 |  | QST cold detection threshold (value) |  | 0.94 (0.89-1.00) | 0.062 |
| IENFD (fibers/mm) |  | 0.75 (0.63-0.88) | <0.001 |  | IENFD (fibers/mm) |  | 0.79 (0.66-1.00) | 0.014 |
| IENFD ≥4.2 | 5/33 (15.2%) | reference |  |  | IENFD ≥1.0 | 9/107 (8.4%) | reference |  |
| IENFD <4.2 | 15/22 (68.2%) | 9.26 (3.04-28.25) | <0.001 |  | IENFD <1.0 | 7/17 (41.2%) | 5.88 (2.17-15.92) | <0.001 |
| P value | <0.001 |  |  |  | P value | 0.001 |  |  |
|  |  |  |  |  |  |  |  |  |
| Multivariable |  | HR (95%-CI) | P value |  | Multivariable |  | HR (95%-CI) | P value |
| Age (per 5 years) |  | 1.54 (1.02-2.33) | 0.041 |  | Age (per 5 years) |  | 1.38 (1.09-1.74) | 0.008 |
| Male vs. Female |  | 2.46 (0.41-14.80) | 0.325 |  | Male vs. Female |  | 1.65 (0.38-7.14) | 0.507 |
| QST cold detection threshold (value) |  | 0.90 (0.82-1.00) | 0.046 |  | QST cold detection threshold (value) |  | 1.00 (0.91-1.10) | 0.985 |
| IENFD ≥4.2 |  | reference |  |  | IENFD ≥1.0 |  | reference |  |
| IENFD <4.2 |  | 6.20 (1.73-22.24) | 0.005 |  | IENFD <1.0 |  | 3.74 (0.83-16.84) | 0.085 |
| *Age, sex, QST cold detection threshold, and IENFD<4.2 were entered as covariates into the multivariable Cox regression model using the "Enter" method.* | | | |  | *Age, sex, QST cold detection threshold, and IENFD<1.0 were entered as covariates into the multivariable Cox regression model using the "Enter" method.* | | | |

**Table 7s:** Cox regression model including QST cold detection threshold

| **Model** | **Covariates in model** | **HR (95%-CI)** | **P value** | **-2LL** | **Δ−2LL vs. previous block** | **AICc** | **BIC** |
| --- | --- | --- | --- | --- | --- | --- | --- |
| Base A | Age (per 5 years) | 1.91 (1.33-2.74) | <.001 | 109.83 | - | 117.33 | 118.82 |
|  | Male vs. female | 7.13 (1.45-35.01) | 0.015 |  |  |  |  |
|  | QST cold detection | 0.93 (0.85-1.01) | 0.081 |  |  |  |  |
|  |  |  |  |  |  |  |  |
| Base A  + IENFD <4.2 | Age (per 5 years) | 1.54 (1.02-2.33) | 0.041 | 100.58 | 9.25  (p=0.002) | 111.25 | 112.57 |
|  | Male vs. female | 2.46 (0.41-14.80) | 0.325 |  |  |  |  |
|  | QST cold detection | 0.90 (0.82-1.00) | 0.046 |  |  |  |  |
|  | IENFD<4.2 | 6.20 (1.73-22.24) | 0.005 |  |  |  |  |
|  |  |  |  |  |  |  |  |
| Base B | Age (per 5 years) | 1.79 (1.22-2.63) | 0.003 | 104.68 | - | 112.18 | 113.67 |
|  | Male vs. female | 6.44 (1.43-29.09) | 0.015 |  |  |  |  |
|  | IENFD<4.2 | 5.26 (1.53-18.03) | 0.008 |  |  |  |  |
|  |  |  |  |  |  |  |  |
| Base B  + QST cold detection | Age (per 5 years) | 1.54 (1.02-2.33) | 0.041 | 100.58 | 4.09  (p=0.043) | 111.25 | 112.57 |
|  | Male vs. female | 2.46 (0.41-14.80) | 0.325 |  |  |  |  |
|  | IENFD <4.2 | 6.20 (1.73-22.24) | 0.005 |  |  |  |  |
|  | QST cold detection | 0.90 (0.82-1.00) | 0.046 |  |  |  |  |
|  |  |  |  |  |  |  |  |

**Table 8s:** Nested cox models for incident LGE (events=20; analyzed n=54)

Abbreviations: **AICc** = Akaike information criterion (small-sample corrected); **BIC** = Bayesian information criterion; **CI** = confidence interval; **df** = degrees of freedom; **HR** = hazard ratio; **IENFD** = intraepidermal nerve fiber density; **LGE** = late gadolinium enhancement; **QST** = quantitative sensory testing; **−2LL** = minus two log-likelihood.

| **Model** | **Covariates in model** | **HR (95% CI)** | **p value** | **−2LL** | **Δ−2LL vs. previous block** | **AICc** | **BIC** | |
| --- | --- | --- | --- | --- | --- | --- | --- | --- |
| Base A | Age (per 5 years) | 1.43 (1.14–1.78) | 0.002 | 119.23 | - | 127.23 | 127.55 | |
|  | Sex (male vs. female) | 2.40 (0.66–8.69) | 0.183 |  |  |  |  | |
|  | QST cold detection | 0.97 (0.89–1.04) | 0.319 |  |  |  |  | |
|  |  |  |  |  |  |  |  | |
| Base A +  IENFD <1.0 | Age (per 5 years) | 1.38 (1.09–1.74) | 0.008 | 116.08 | 3.15  (p=0.076) | 127.72 | 127.17 | |
|  | Sex (male vs. female) | 1.65 (0.38–7.14) | 0.507 |  |  |  |  | |
|  | QST cold detection | 1.00 (0.91–1.10) | 0.985 |  |  |  |  | |
|  | IENFD <1.0 (vs. ≥1.0) | 3.74 (0.83–16.84) | 0.085 |  |  |  |  | |
|  |  |  | |  |  |  |  |  |
| Base B | Age (per 5 years) | 1.38 (1.09–1.74) | 0.008 | 116.08 | - | 124.08 | 124.40 | |
|  | Sex (male vs. female) | 1.64 (0.41–6.56) | 0.487 |  |  |  |  | |
|  | IENFD <1.0 (vs. ≥1.0) | 3.72 (1.00–13.78) | 0.050 |  |  |  |  | |
|  |  |  |  |  |  |  |  | |
| Base B +  QST cold detection | Age (per 5 years) | 1.38 (1.09–1.74) | 0.008 | 116.08 | 0.00  (p=0.985) | 127.72 | 127.17 | |
|  | Sex (male vs. female) | 1.65 (0.38–7.14) | 0.507 |  |  |  |  | |
|  | IENFD <1.0 (vs. ≥1.0) | 3.74 (0.83–16.84) | 0.085 |  |  |  |  | |
|  | QST cold detection | 1.00 (0.91–1.10) | 0.985 |  |  |  |  | |

**Table 9s:** Nested cox models for ICD/pacemaker implantation (events = 16; analyzed n = 123)

Abbreviations: **AICc** = Akaike information criterion (small-sample corrected); **BIC** = Bayesian information criterion; **CI** = confidence interval; **df** = degrees of freedom; **HR** = hazard ratio; **ICD** = implantable cardioverter-defibrillator; **IENFD** = intraepidermal nerve fiber density; **QST** = quantitative sensory testing; **−2LL** = minus two log-likelihood.

**Results from nested Cox models showed the following key aspects for the first endpoint (development of LGE in MRI):**

Adding QST cold detection to a model with IENFD<4.2 modestly but significantly improved fit (Δ−2LL=4.10, p=0.043) with small information-criterion gains (ΔAICc=−0.93; ΔBIC=−1.10). By adding IENFD <4.2 to a model with QST produced a larger improvement (Δ−2LL=9.25, p=0.002) and stronger information-criterion gains (ΔAICc=−6.08; ΔBIC=−6.25). In the joint model, both markers were independently associated with LGE; however, the larger Δ−2LL and greater AICc/BIC reductions when adding IENFD indicate stronger incremental value from IENFD.

In nested Cox models adjusted for age and sex, both IENFD and QST (cold detection value) contributed independent information for incident LGE. Starting from the QST base model (age, sex, QST), adding IENFD<4.2 fibers/mm markedly improved fit (Δ−2LL=9.25, p=0.002) and IENFD<4.2 was independently associated with higher risk (HR=6.20, 95%-CI 1.73–22.24, p=0.005); QST remained significant (HR per unit=0.90, 95%-CI 0.82–1.00, p=0.046), and age was retained (per 5 years: HR=1.54, 95%-CI 1.02–2.33, p=0.041), while sex was not (p=0.325). Conversely, starting from the IENFD base model (age, sex, IENFD<4.2), adding QST yielded a smaller(yet significant)improvement (Δ−2LL=4.10, p=0.043). Information criteria were concordant: adding IENFD to the QST base model reduced AICc and BIC from 117.33 to 111.25 and from 118.82 to 112.57, respectively (ΔAICc=−6.08; ΔBIC=−6.25), whereas adding QST to the IENFD based model produced smaller reductions (AICc 112.18→111.25; BIC 113.67→112.57; ΔAICc=−0.93; ΔBIC=−1.10).

Overall, IENFD conferred the larger incremental improvement, with QST providing complementary—but more modest—prognostic information for incident LGE.

**Results from nested Cox models showed the following key aspects for the second endpoint (device implantation):**

Starting from a base model with age, sex, and QST cold detection (Base A), adding IENFD<1.0 fibers/mm improved fit numerically (Δ−2LL=3.15, p=0.076) and slightly reduced BIC (127.55→127.17), although AICc increased (127.23→127.72). In this expanded model, IENFD showed a three- to fourfold higher hazard that did not reach conventional significance (HR=3.74, 95% CI 0.83–16.84, p=0.085), while QST was not associated (p=0.985). Conversely, starting from a base model with age, sex, and IENFD<1.0 (Base B), adding QST did not improve fit (Δ−2LL=0.00, p=0.985) and worsened both AICc and BIC (124.08→127.72; 124.40→127.17). In the Base B model, IENFD<1.0 was associated with increased risk at the threshold of significance (HR=3.72, 95%-CI 1.00–13.78, p=0.050). Overall, information-criterion comparisons favored the age + sex + IENFD model (lowest AICc/BIC), and nested tests indicated no incremental value of QST once IENFD was included, supporting IENFD as the more informative marker for device-therapy risk in this cohort.

Overall, we elected to exclude quantitative sensory testing (QST) from the final prediction models. For the first endpoint, intraepidermal nerve fiber density (IENFD) demonstrated superior predictive performance for late gadolinium enhancement (LGE) on MRI, yielding a more robust model than that incorporating QST. Regarding the second endpoint, the initial model excluding QST was favored, as nested model comparisons indicated no significant improvement when QST was added alongside IENFD.

| **Cox regression models for predicting LGE development in FD patients** | | | |  | **Cox regression models for predicting device implantation** | | | |
| --- | --- | --- | --- | --- | --- | --- | --- | --- |
| Univariable |  | HR (95%-CI) | P value |  | Univariable |  | HR (95%-CI) | P value |
| Age (per 5 years) |  | 1.35 (1.09-1.72) | 0.016 |  | Age (per 5 years) |  | 1.35 (1.1-1.67) | 0.005 |
| Male vs. Female |  | 2.1 (0.85-5.2) | 0.108 |  | Male vs. Female |  | 1.83 (0.7-4.83) | 0.225 |
| IENFD (per 1 fiber/mm increase) |  | 0.75 (0.63-0.88) | <0.001 |  | IENFD (per 1 fiber/mm increase) |  | 0.8 (0.66-0.96) | 0.014 |
|  |  |  |  |  |  |  |  |  |
| Multivariable |  | HR (95% CI) | P value |  | Multivariable |  | HR (95% CI) | P value |
| Age (per 5 years) |  | 1.8 (1.21-2.67) | 0.004 |  | Age (per 5 years) |  | 1.33 (1.05-1.69) | 0.018 |
| Male vs. Female |  | 7.02 (1.41-35.02) | 0.018 |  | Male vs. Female |  | 1.820 (0.52-6.31) | 0.346 |
| IENFD (per 1 fiber/mm increase) |  | 0.86 (0.71-1.04) | 0.119 |  | IENFD (per 1 fiber/mm increase) |  | 0.87 (0.71-1.08) | 0.218 |
| *Age, sex, and IENFD were entered as covariates into the multivariable Cox regression model using the "Enter" method.* | | | |  | *Age, sex, and IENFD were entered as covariates into the multivariable Cox regression model using the "Enter" method.* | | | |

**Table 10s:** Sensitivity analyses modeling IENFD as a continuous variable

Hazard ratios (HRs) are presented with 95% confidence intervals (CI). IENFD was modeled as a continuous variable (per 1 fiber/mm increase). Multivariable Cox regression models were fitted using the enter method and adjusted for age and sex.

**Sensitivity analyses using continuous IENFD**

To address potential non-linearity and information loss due to dichotomization, sensitivity analyses were performed modeling IENFD as a continuous variable. In univariable Cox regression, lower IENFD was significantly associated with both incident LGE and cardiac device implantation. After adjustment for age and sex, these linear associations were attenuated and no longer statistically significant in multivariable models (Table 10s). This pattern suggests that the prognostic relevance of IENFD is not strictly linear across its full range but is primarily driven by advanced stages of skin denervation. Accordingly, threshold-based IENFD categories may better capture clinically meaningful cardiac risk, while continuous modeling confirms the overall direction of association.

To address potential heterogeneity between brady- and tachycardiac indications, device implantation was examined in detail, and event rates are reported separately for pacemaker and ICD implantation in the Supplement (Table 2S).

Pacemaker implantation (n=5) occurred predominantly for bradycardiac indications (symptomatic bradycardia or atrioventricular block) and was associated with reduced IENFD. ICD implantation (n=12) was mainly driven by VTs or primary prophylactic indications and occurred in patients with markedly lower IENFD values.

In ICD-only analyses, lower IENFD was associated with subsequent ICD implantation in univariable Cox regression, and this relationship is illustrated by cumulative hazard curves derived from the univariable model (Figure 2s). After adjustment for age, effect estimates were attenuated, likely reflecting the limited number of events. Given the small number of prophylactic ICD implantations, these patients were retained within the ICD endpoint, and no additional stratified time-to-event analyses were performed to avoid further loss of statistical power. Notably, only three such events occurred, and the corresponding IENFD values were comparatively higher (6.6 [3.3–7.9]), indicating that these cases did not materially influence or drive the observed association between IENFD and outcome in the way that it improved IENFD as a prognostic marker.

**
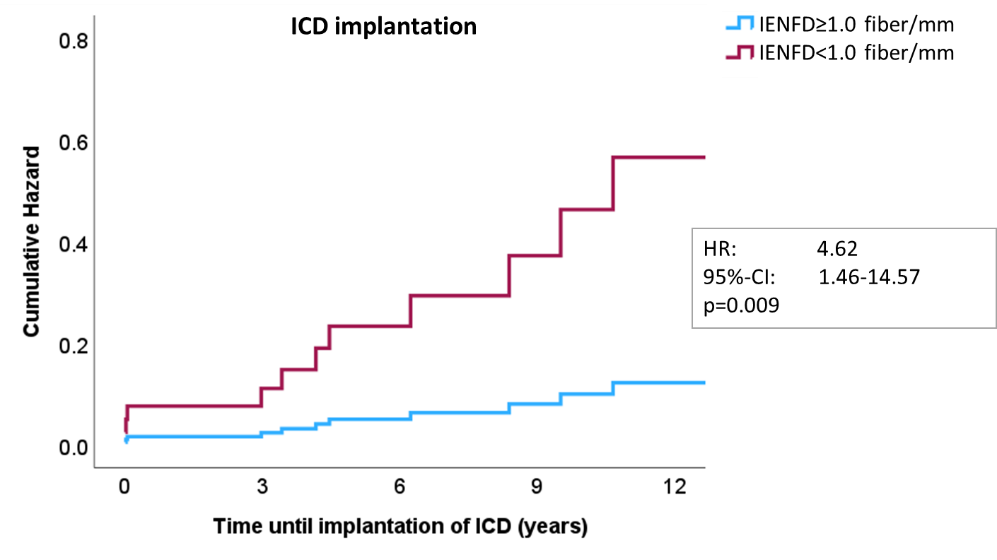
**

**Figure 2s:** Cumulative hazard of ICD implantation according to IENFD

Cumulative hazard curves for ICD implantation derived from a univariable Cox proportional hazards model, stratified by IENFD (<1.0 vs ≥1.0 fiber/mm). Patients with IENFD <1.0 fiber/mm showed a higher cumulative hazard of ICD implantation during follow-up compared with those with preserved IENFD. Hazard ratio (HR) and 95% confidence interval (CI) are shown in the figure.

| **A. Endpoint: Incident late gadolinium enhancement (LGE) on cardiac MRI** | | | | | | |
| --- | --- | --- | --- | --- | --- | --- |
| **Model** | **Added covariate (tested one-at-a-time)** | **IENFD** | **IENFD HR (95%-CI)** | **P value** | **Added covariate HR (95%-CI)** | **P value** |
| **A0** | None (base model) | IENFD <4.2 vs. ≥4.2 | 5.26 (1.53–18.06) | 0.008 | - | - |
| **A1** | eGFR (per 10 ml/min/1.73m²) | IENFD <4.2 vs. ≥4.2 | 5.15 (1.51-17.54) | 0.009 | 1.06 (0.84-1.32) | 0.631 |
| **A2** | FD-specific therapy (yes vs. no) | IENFD <4.2 vs. ≥4.2 | 5.43 (1.57-18.83) | 0.008 | 1.37 (0.4-4.72 | 0.616 |
| **A3** | Phenotype (classic vs. late-onset) | IENFD <4.2 vs. ≥4.2 | 3.39 (0.9-12.68) | 0.070 | 6.6 (0.66-66.25) | 0.109 |
| **A4** | Hs-TNT (log-transformed) | IENFD <4.2 vs. ≥4.2 | Not estimable* |  |  |  |
| **A5** | NT-proBNP (log-transformed) | IENFD <4.2 vs. ≥4.2 | 8.23 (2.11-32.12) | 0.002 | 10.407 (1.84-59) | 0.008 |
|  |  |  |  |  |  |  |
| **B. Endpoint: Cardiac device implantation (ICD or pacemaker)** | | | | | | |
| **Model** | **Added covariate (tested one-at-a-time)** | **IENFD** | **IENFD HR (95%-CI)** | **P value** | **Added covariate HR (95%-CI)** | **P value** |
| **B0** | None (base model) | IENFD <1.0 vs ≥1.0 | 3.72 (1.0–13.78) | 0.050 | - | - |
| **B1** | eGFR (per 10 ml/min/1.73m²) | IENFD <1.0 vs ≥1.0 | 4.41 (1.12-17.24) | 0.033 | 0.97 (0.83-1.13) | 0.707 |
| **B2** | FD-specific therapy (yes vs. no) | IENFD <1.0 vs ≥1.0 | 3.12 (0.84-11.67) | 0.091 | 4.53 (0.56-36.51) | 0.156 |
| **B3** | Phenotype (classic vs. late-onset) | IENFD <1.0 vs ≥1.0 | 3.59 (0.92-14.03) | 0.066 | 0.94 (0.23-3.84) | 0.928 |
| **B4** | Hs-TNT (log-transformed) | IENFD <1.0 vs ≥1.0 | 3.31 (0.34-31.9) | 0.300 | 9.98 (1.04-95.7) | 0.046 |
| **B5** | NT-proBNP (log-transformed) | IENFD <1.0 vs ≥1.0 | 2.27 (0.54-9.32) | 0.269 | 6.94 (1.81-26.66) | 0.005 |
|  |  |  |  |  |  |  |
|  |  |  |  |  |  |  |

**Table 11s:** Sensitivity multivariable Cox regression models adjusting for additional clinical covariates

Cox proportional hazards models. The base model included age, sex, and IENFD using threshold-based parameterization (LGE: <4.2 vs ≥4.2 fiber/mm; device implantation: <1.0 vs ≥1.0 fiber/mm). Sensitivity analyses were performed by adding one additional covariate at a time to the base model to minimize overparameterization. Renal function was modeled as eGFR per 10 ml/min/1.73 m²; phenotype was categorized as classic versus late-onset; NT-proBNP and high-sensitivity troponin T (hs-TnT) were log-transformed.

* Models including hs-TnT were unstable due to sparse events and were therefore not interpreted.

**Sensitivity analyses with additional clinical covariates**

To assess whether the association between IENFD and cardiac outcomes was influenced by other clinically relevant factors, prespecified sensitivity analyses were performed in which renal function, FD-specific therapy status, phenotype (classic vs late-onset), and NT-proBNP were each added individually to the base Cox model including age, sex, and IENFD. For incident LGE, the association between low IENFD and outcome remained directionally consistent across sensitivity models and remained statistically significant after adjustment for NT-proBNP (Table 11s-A). For cardiac device implantation, effect estimates for IENFD were similarly directionally consistent but attenuated in some models, reflecting the limited number of events (Table 11s-B). Overall, these findings support the robustness of the association between advanced skin denervation and cardiac outcomes while acknowledging limited power for fully adjusted models.
